# Influence of pterygium size on corneal higher-order aberration evaluated using anterior-segment optical coherence tomography

**DOI:** 10.1186/s12886-018-0837-8

**Published:** 2018-07-09

**Authors:** Keiichiro Minami, Tadatoshi Tokunaga, Keiichiro Okamoto, Kazunori Miyata, Tetsuro Oshika

**Affiliations:** 1 Miyata Eye Hospital, 6-3 Kurahara-cho, Miyakonojo, Miyazaki, 885-0051 Japan; 2 Tomey Corporation, Nishi-ku, Nagoya, Aichi Japan; 3 Department of Ophthalmology, Faculty of Medicine, University of Tsukuba, Tsukuba, Ibaraki, Japan

**Keywords:** Pterygium, Anterior-segment optical coherence tomography, Zernike analysis, Higher-order aberration

## Abstract

**Background:**

The prospective observation study aimed to evaluate changes in corneal higher-order aberrations induced by advancement of pterygium using an anterior-segment optical coherence tomography (AS-OCT) and Zernike aberration analysis.

**Methods:**

The corneal topography of 284 eyes with primary pterygia originating from the nasal region was measured using an AS-OCT (SS-1000, Tomey). With anterior corneal elevation data, Zernike polynomial coefficients were calculated in diameters of 1.0, 3.0, and 5.0 mm, and the coma, spherical, coma-like, spherical-like, and total higher-order aberrations were obtained. Pterygium size was also measured as a ratio of positions of the pterygium end with respect to the corneal diameter and categorized in eight classes: less than 15%, 15–20%, 20–25%, 25–30%, 30–35%, 35–40%, 40–45, and 45% or larger. Increases in the aberrations were analyzed with reference to those in eyes with pterygium size < 15%.

**Results:**

The mean age of the participants was 69.3 years, and the pterygium size ranged from 2 to 57% (mean: 28.8%). The coma aberration significantly increased when the pterygium size was 45% or larger in 1.0 and 3.0 mm diameters and over 25–30% in 5.0 mm diameter. Similar increases were found in the pterygium sizes exceeding 45, 40, and 25%, respectively, in the coma-like, spherical-like, and total higher-order aberrations. On contrast, there was no increase in the spherical aberration.

**Conclusion:**

Increases in higher-order aberrations reflected the pterygium size, and significant aberrations were induced in 5.0 mm diameter when the end exceeded 25% of corneal diameter. The use of AS-OCT and Zernike analysis could enable objective grading of pterygium advancement based on changes in corneal optics.

**Electronic supplementary material:**

The online version of this article (10.1186/s12886-018-0837-8) contains supplementary material, which is available to authorized users.

## Background

Pterygium, the growth of conjunctival tissue in the cornea, induces topographical irregularity. Consequently, the surface regularity index (SRI) [1, 2], higher-order irregularity (HOI) in Fourier harmonic analysis of topographic data [3, 4], and higher-order Zernike coefficients [5, 6] increase with the advancement of a pterygium. Increased corneal irregularity leads to degradation of contrast sensitivity as well as visual impairment. Such increases could reduce the benefits of premier intraocular lens implantation [7, 8], whereas corneal irregularity has been evaluated in a particular diameter [1–6]. Hence, we modified the Fourier analysis of Placido topography data and evaluated changes in the HOI due to primary pterygia in diameters of 1.0–8.0 mm [9]. The analysis revealed steep increases in the HOI when the end of pterygium is close to the analysis diameter. With the use of this objective evaluation of corneal irregularity, the severity of pterygium could be graded [4].

**Fig. 1:**
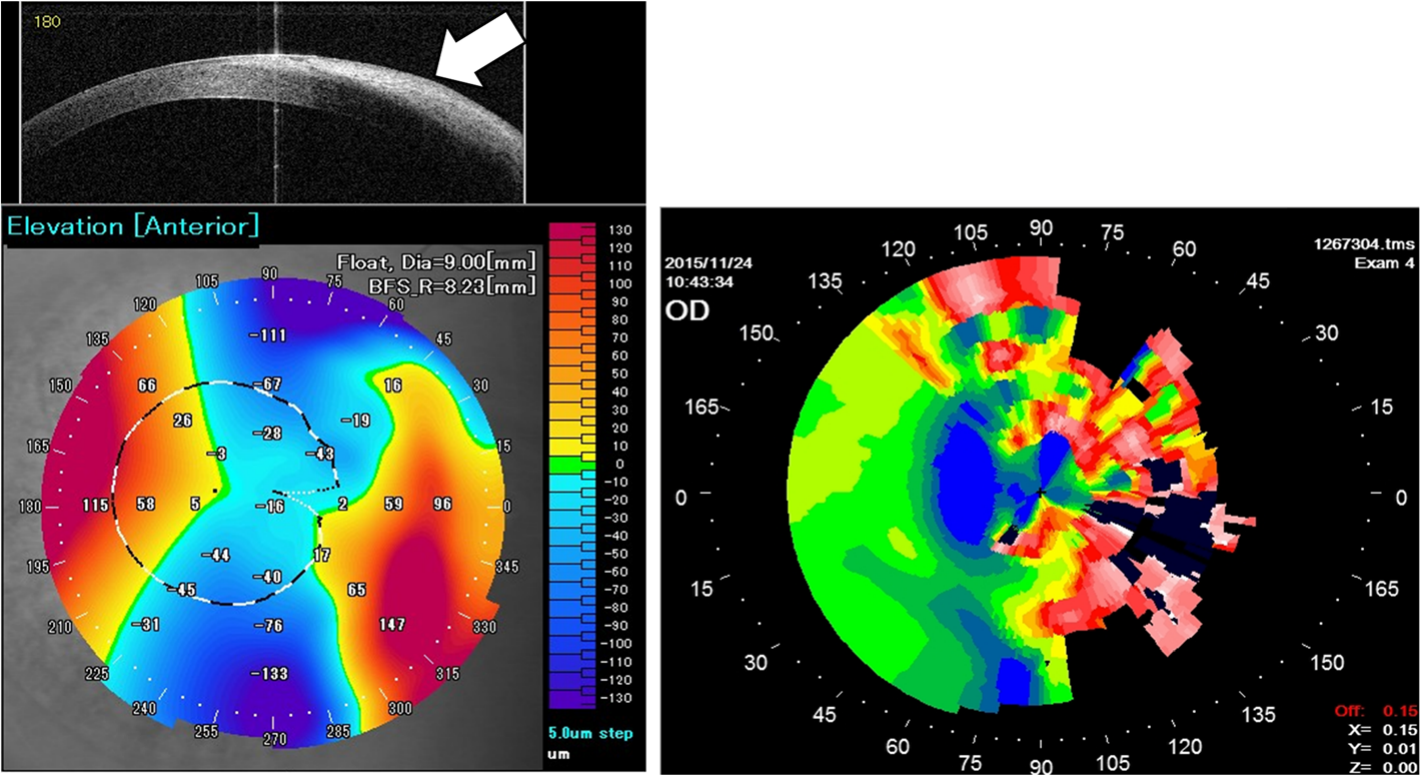
Topographic maps of an eye with the pterygium measured by AS-OCT (left) and Placido (right) topographers. The use of AS-OCT allowed full topographic mapping without obvious defects while the pterygium advanced up to the corneal apex

The use of anterior-segment optical coherence tomography (AS-OCT) enables accurate measurement of the anterior corneal elevation of abnormal eyes [10]. Figure 1 is topographic maps of an eye with the pterygium advancing to the center of the cornea, measured by the AS-OCT and Placido topographers. Although the pterygium surface could not be projected by Mire-ring images, the AS-OCT allowed topographic mapping without obvious defects. In the previous analysis using Placido topography [4, 5], poor quality measurements due to highly irregular surfaces were observed, resulting in incomplete topography maps. In contrast, the use of AS-OCT is more suitable for the pterygium surface. The measured AS-OCT topography could be analyzed using the Zernike polynomial expansion [11], which is more representative of optical aberrations of the cornea.

The study aimed to evaluate increases in corneal higher-order aberrations induced by advancement of pterygium using an AS-OCT and Zernike aberration analysis in multiple diameters.

## Methods

The protocol of this prospective observation was approved by the ethics committee of Miyata Eye Hospital (identifier: CS-231-036), and the study adhered to the tenets of the Declaration of Helsinki. Patients who underwent pterygium excision surgery from July 2014 to December 2016 at Miyata Eye Hospital due to primary pterygium were recruited. Written informed consent for the use of clinical data was obtained from all patients before examinations. Inclusion criteria were primary pterygium originating from the nasal area without a history of any surgical treatment. Eyes that used contact lenses or had corneal diseases influencing the corneal topography such as keratoconus, primary irregular astigmatism, and corneal degeneration, were excluded.

The study comprised 284 eyes from 242 patients, and the ages of the patients ranged from 35 to 92 years (mean: 69.3 years). There were 5 pseudophakic eyes, and 68 eyes were planned for cataract surgery after pterygium excision. Preoperatively, anterior corneal topography was measured using an AS-OCT (SS-1000, Tomey). Zernike coefficients up to the sixth order were calculated from the anterior corneal elevation map data [5, 6], using the topography viewer software that was modified for calculations in diameters of 1.0 to 6.0 mm with a step of 1.0 mm (ASOCT viewer ver. 4.8.4 M3, Tomey). Coma, spherical, coma-like, spherical-like, and higher-order aberrations were obtained. The amplitude of coma aberration was calculated as an absolute amplitude of the relevant vertical and horizontal components. The coma-like and spherical-like aberrations were root mean squares (RMS) of the fourth- and sixth-order coefficients and the third- and fifth-order coefficients, respectively. Higher-order aberration was RMS of the third to sixth orders.

Ocular images were captured with a digital camera and a ratio of positions of the pterygium end with respect to the corneal diameter was obtained as pterygium size (%) [2–4]. Best corrected visual acuity (BCVA) was examined and converted to logarithm of minimum angle of resolution (logMAR) for analysis.

### Statistical analysis

Pterygia normally originate on the nasal side; hence, it was supposed that asymmetry irregularity would be increased with the pterygium size. For examining this assumption, changes in the coma and spherical aberrations with the pterygium size were compared for the diameters of 1.0, 3.0, and 5.0 mm [4]. Evaluation of a 1.0 mm diameter was considered the severest case, with a significant risk of visual function degradation. Analysis of a 3.0-mm diameter was relevant with photopic pupil diameter in adults or older adults that ranges from 2.20 to 3.77 mm [12]. This analysis diameter also has been used in conventional irregularity analysis such as SRI [1, 2]. The use of a 5.0-mm diameter was for examining the effect on the mesopic contrast sensitivity [7]. Pterygium sizes were divided into eight classes: less than 15% (< 15%), 15–20%, 20–25%, 25–30%, 30–35%, 35–40%, 40–45, and 45% or larger (≥ 45%).

**Fig. 2:**
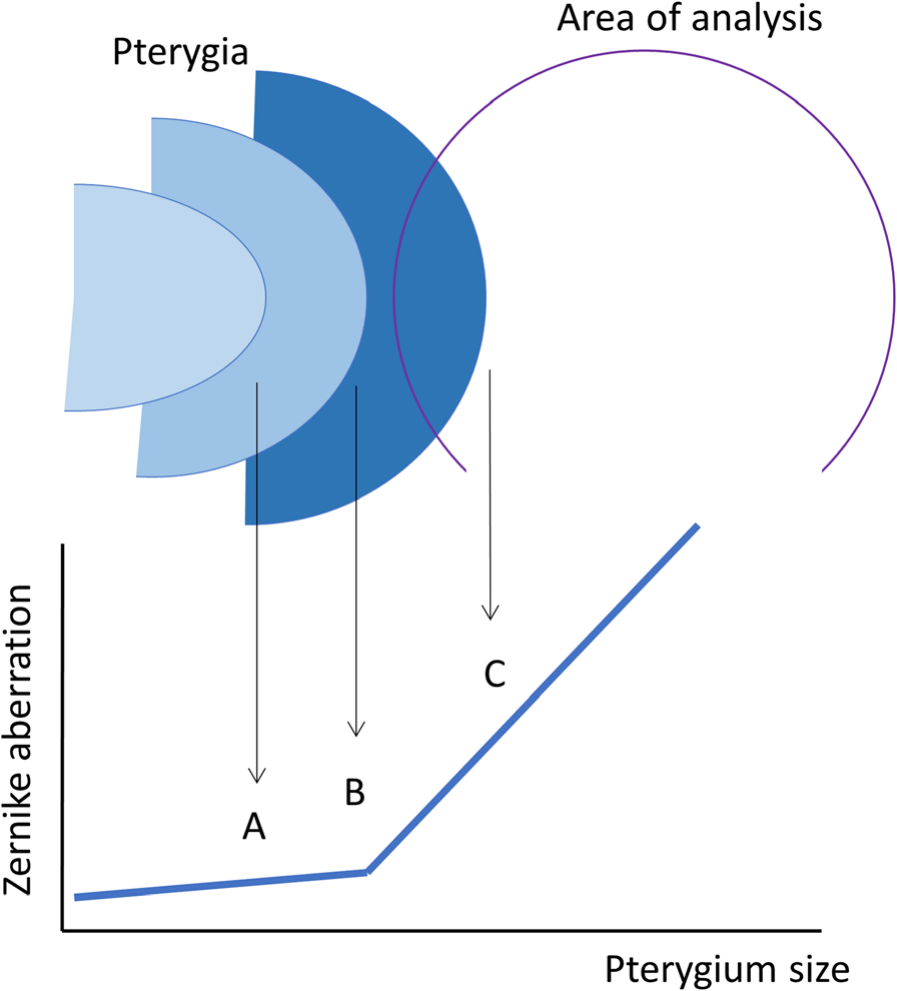
Pterygium size and change in Zernike aberrations. In eyes with small pterygium, Zernike aberration of the corneal surface (point A). As the pterygium end is close to or within the area of Zernike analysis (points B and C, respectively), surface irregularity induced by the advancement could increase Zernike aberrations

When pterygium was sufficiently small and the end was not close to area of Zernike analysis, the aberration obtained should represent the corneal surface. As the pterygium end was close to the analysis area, surface irregularity induced by the advancement could increase Zernike aberrations (Fig. 2). Changes in the aberrations with the pterygium size classes were evaluated using one-way ANOVA. If the change was significant (*P* < 0.05), the differences from the values of < 15%, in which pterygium could least influence the cornea, were examined using the Dunnet multiple comparison. In addition, difference between the coma and spherical aberrations were evaluated using a paired *t*-test. The coma-like and spherical-like aberrations were also compared in the same manner. For the higher-order aberration, changes in the pterygium size were evaluated.

The changes in the BCVA with the pterygium sizes were examined. After excluding eyes with ocular diseases influencing visual acuity such as cataract, the BCVA for the 8 pterygium sizes were compared using the Kruskal-Wallis test following the Steel-Dwass multiple comparison.

Two-tailed *P* < 0.05 was considered a significant difference. Results are expressed as mean ± standard deviation.

## Results

The mean pterygium size was 28.8 ± 10.5%, ranging from 2 to 57%. There were 18, 25, 78, 57, 28, 24, 26, and 28 eyes in the pterygium sizes of < 15%, 15–20%, 20–25%, 25–30%, 30–35%, 35–40%, 40–45%, and ≥ 45%, respectively. The mean Zernike aberrations for 1.0, 3.0, and 5.0 mm diameters are shown in Table 1. The coma aberrations were higher than the spherical aberrations in 3.0 and 5.0 mm diameters (*P* < 0.001). On the other hand, the coma-like was less than the spherical-like in diameters of 1.0 mm and vice versa in 5.0 mm. All aberrations increased with the pterygium size (*P* < 0.01, linear regression analysis) except for the spherical aberration in 5.0 mm diameter (*P* = 0.083).

**Table 1:** Zernike aberrations in 1.0, 3.0, and 5.0 mm diameters

Diameter	1.0 mm	3.0 mm	5.0 mm
Coma aberration (μm)	0.07 ± 0.09	0.30 ± 0.54	1.08 ± 1.21
Spherical aberration (μm)	0.10 ± 0.16	0.10 ± 0.28	−0.02 ± 0.53
*P* value	0.0069	< 0.001	< 0.001
Coma-like aberration (μm)^a^	0.21 ± 0.33	0.53 ± 0.89	2.43 ± 2.56
Spherical-like aberration (μm)^a^	0.27 ± 0.42	0.49 ± 0.87	1.63 ± 1.76
*P* value	< 0.001	0.14	< 0.001
Higher-order aberration (μm)^a^	0.35 ± 0.53	0.74 ± 1.24	2.95 ± 3.09

^a^: RMS values

**Fig. 3:**
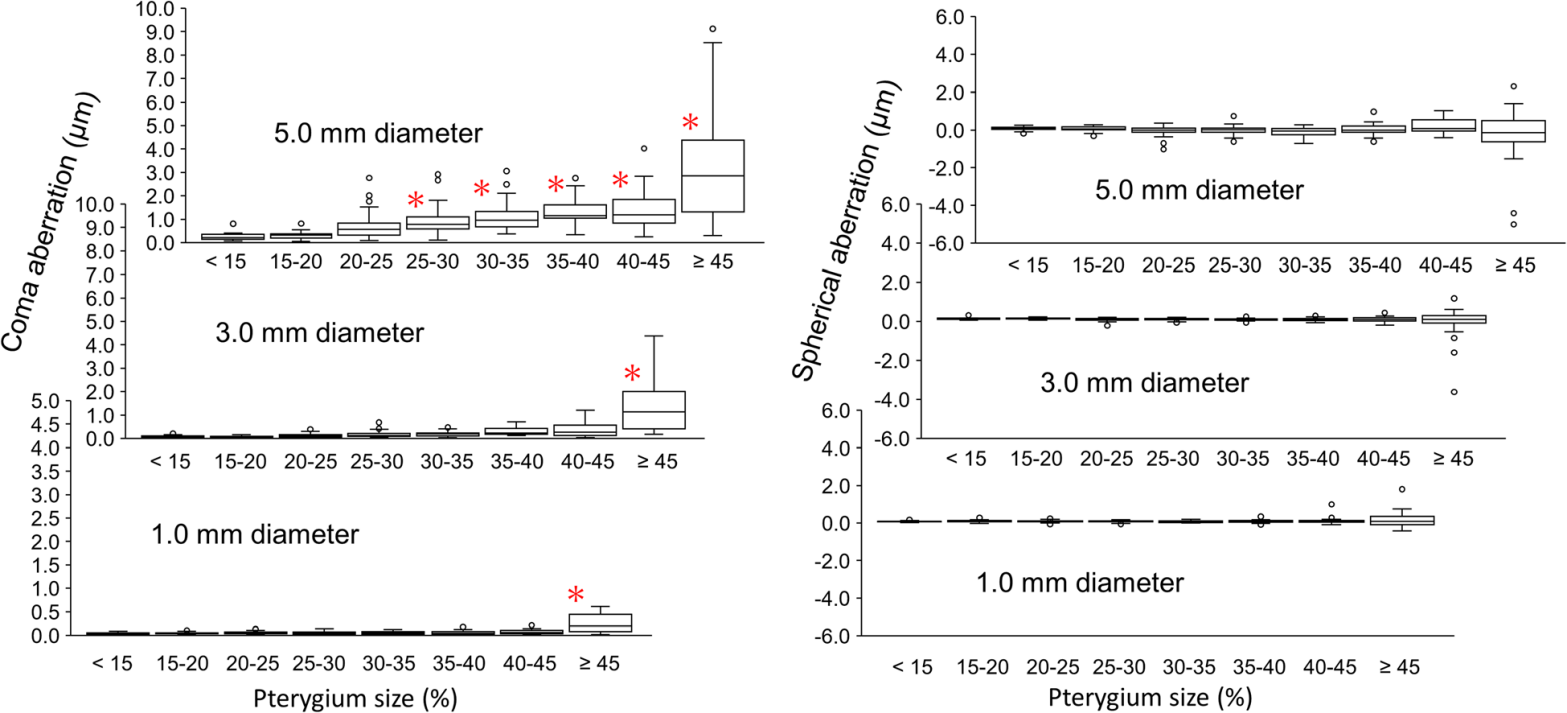
Changes in the coma (left side) and spherical (right side) aberrations in diameters of 1.0, 3.0, and 5.0 mm with pterygium sizes. * denotes significant difference from the values with the pterygium size below 15% (the Dunnet multiple comparison)

Figure 3 shows changes in the coma and spherical aberrations in 1.0, 3.0, and 5.0 mm diameters with the pterygium sizes. The coma aberrations in the 1.0 and 3.0 mm diameters (lower and middle left) were significantly different from those of the pterygium size of < 15%, when the pterygium size was ≥45% (*P* < 0.001). There was significant increase with the pterygium size of 25–30% or larger (*P* < 0.037) in the 5.0 mm diameter (upper left). In the spherical aberrations (right side), such a significant increase was not found (*P* > 0.05).

**Fig. 4:**
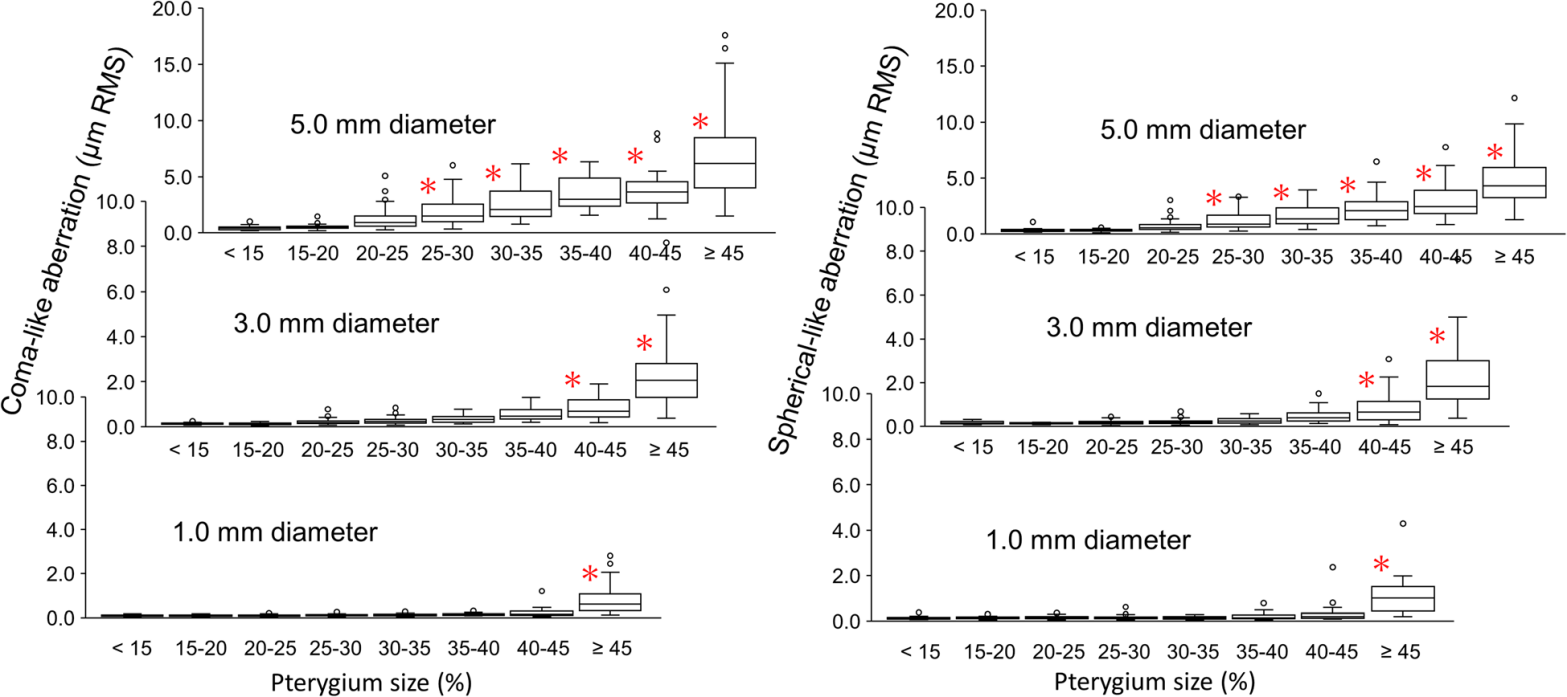
Changes in the coma-like (left side) and spherical-like (right side) aberrations in diameters of 1.0, 3.0, and 5.0 mm with pterygium sizes. * denotes significant difference from the values with the pterygium size below 15% (the Dunnet multiple comparison)

Figure 4 shows changes in the coma-like and spherical-like aberrations. In both aberrations, significant difference from the pterygium size of < 15% was found when the pterygium size was ≥45% in the 1.0 mm diameter (*P* < 0.001) and 40–45% or larger in the 3.0 mm diameter (*P* < 0.0034). In the 5.0 mm diameter, significant increases were found with the pterygium size of 25–30% or larger (*P* < 0.0033).

**Fig. 5:**
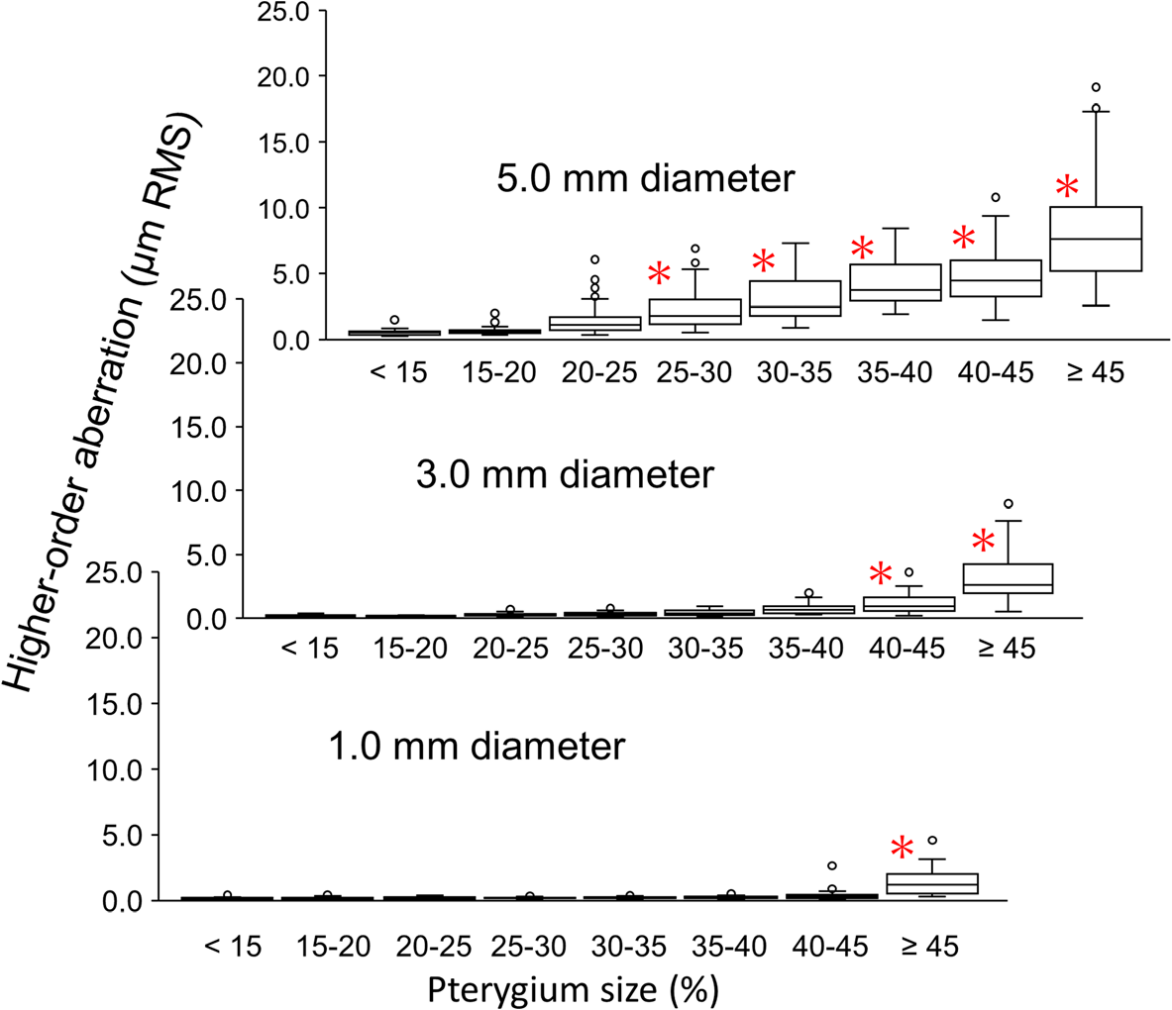
Changes in the higher-order aberration in diameters of 1.0, 3.0, and 5.0 mm with pterygium sizes. * denotes significant difference from the values with the pterygium size below 15% (the Dunnet multiple comparison)

Figure 5 shows changes in the higher-order aberrations. Significant differences from the pterygium size of < 15% were found in the same manner as for the coma-like and spherical-like aberrations: the pterygium sizes of ≥45%, 40–45% or larger, and 25–30% or larger in the diameters of 1.0, 3.0, and 5.0 mm (*P* < 0.001, 0.0016, and 0.011), respectively.

The BCVA were analyzed in 201 eyes. The mean logMAR BCVA in the pterygium sizes of < 15%, 15–20%, 20–25%, 25–30%, 30–35%, 35–40%, 40–45%, and ≥ 45% were 0.01 ± 0.29, − 0.04 ± 0.13, 0.09 ± 0.35, 0.02 ± 0.13, 0.00 ± 0.14, 0.13 ± 0.28, 0.12 ± 0.25, and 0.28 ± 0.36, respectively. The BCVA in the pterygium size of ≥ 45% was significantly worse than the other sizes (*P* > 0.01, Steel-Dwass multiple test).

## Discussion

The coma aberration showed significant increases when the pterygium size was over 45% in 1.0 and 3.0 mm diameters and 25–30% or larger in 5.0 mm diameter, whereas there was no increase in the spherical aberration. The coma-like, spherical-like, and higher-order corneal aberrations in the diameters of 1.0, 3.0, and 5.0 mm were significantly increased when the pterygium size had advanced over ≥45%, 40–45%, and 25–30%, respectively. Pesudovs et al. investigated 67 eyes before pterygium surgery, using a Placido topography, in which the mean higher-order aberrations of a 5.0 mm diameter were 0.94 ± 0.83 μm [5]. The current results were 3.1 times higher. The AS-OCT enables topography measurement for an irregular or abnormal corneal surface [10]. Hence, the current study could analyze the more severe corneal irregularity, resulting in such a difference. Evaluation of 47 preoperative eyes, using Placido and Scheimpflug imaging, resulted in higher-order aberrations of 3.06 ± 2.93 μm in a 6.0 mm diameter [6], which was close to the current results. Ozgurhan et al. reported that the coma, spherical, and higher-order aberrations were correlated with the size of the pterygium [6]. Although the definition of pterygium size and the diameter of the Zernike analysis were not the same, similar trends were obtained in the current study, except for the spherical aberration. The current results showed no significant change in the spherical aberration, and this difference would result from ethnic difference in corneal diameter and the use of AS-OCT topographer.

Corneal irregularity of a primary pterygium was evaluated using Fourier harmonic analysis of Placido topography data within 1.0, 3.0, and 5.0 diameters, and the HOI components steeply increase at the pterygium sizes of 29.7, 21.4, and 16.5%, respectively [4]. Even though the topography measurement technology and the analysis method of corneal irregularity were different, the both results showed that increases in corneal irregularity due to pterygium advancements altered with the diameter of analysis. Hence, it could be convincing that the Zernike analysis in multiple diameters enables an objective evaluation of pterygia based on corneal optical property. From comparison of the pterygium size occurring significant increases, it was demonstrated that the current method was less sensitive to detect pterygium advancement than the previous method.

In the coma-like, spherical-like, and higher-order aberrations, significant increases occurred in the same pterygium sizes. It has been assumed that the third-order aberration mostly contributes to the pterygium size, and the difference from the higher-order aberration is relatively small [5]. The current results also showed that contributions of the coma and coma-like aberrations were higher. Whereas, the spherical-like aberration showed association with the pterygium size, although such an association was not found in the spherical aberration. It was speculated that increases in the pterygium surface (conjunctival epithelium) would increase the fourth-order Zernike coefficients, except for the spherical aberration term. Detailed analysis of the AS-OCT image is necessary to examine the influence of the pterygium surface.

A pterygium grading system was proposed using the Fourier harmonic analysis of Placido topography data [4]. The combination of the AS-OCT and Zernike analysis could detect the pterygium severity in a similar manner. The changes in the BCVA met the significant increases in the coma, coma-like, spherical-like, and higher-order aberration in 1.0 mm diameter. Hence, the ability of AS-OCT in irregular surface measurement, and the Zernike aberration expression that closely represents the optical aberrations, would be more advanced than the previous grading system [4].

There were several limitations in the current study. First, the pterygium size was evaluated in proportion to the corneal diameter. The distance between the pterygium end and the corneal apex was not measured in the current observation study. For more precise evaluation, measurement of the distance from the corneal apex is preferred [13]. Second, although the topography data was obtained with AS-OCT, there were cases in which the topography had partial defects. Although the use of AS-OCT is robust against corneal surface abnormalities [10, 14], measurement of the conjunctival surface on the pterygium is still challenging. Next, the corneal irregularity analysis method depends on the technology used for topography measurement (for example, Placido or AS-OCT). The corneal elevation map is obtained from the Mire ring image in Placido topography, so that radical scale varies with the keratometry. A steep cornea results in a dense rings image and overestimates in the radical scale. Presently, the compatibility is not confirmed.

## Conclusions

The use of AS-OCT and Zernike analysis revealed that significant aberrations were induced in 5.0 mm diameter when the end exceeded 25% of corneal diameter. Such an objective evaluation of corneal higher-order aberration could enable a grading of pterygium advancement based on changes in corneal optics.

## Additional file

Additional file 1: **Table S1.** Dataset of corneal Zernike aberrations of primary pterygia. (XLSX 260 kb)

## Abbreviations

AS-OCT: Anterior-segment optical coherence tomography
HOI: Higher-order irregularity
RMS: Root mean square
SRI: Surface regularity index
